# Nonlinear processing with linear optics

**DOI:** 10.1038/s41566-024-01494-z

**Published:** 2024-07-31

**Authors:** Mustafa Yildirim, Niyazi Ulas Dinc, Ilker Oguz, Demetri Psaltis, Christophe Moser

**Affiliations:** 1 Laboratory of Applied Photonics Devices, Ecole Polytechnique Fédérale de Lausanne (EPFL), Lausanne, Switzerland; 2 Optics Laboratory, Ecole Polytechnique Fédérale de Lausanne (EPFL), Lausanne, Switzerland

**Keywords:** Applied optics, Other photonics

## Abstract

Deep neural networks have achieved remarkable breakthroughs by leveraging multiple layers of data processing to extract hidden representations, albeit at the cost of large electronic computing power. To enhance energy efficiency and speed, the optical implementation of neural networks aims to harness the advantages of optical bandwidth and the energy efficiency of optical interconnections. In the absence of low-power optical nonlinearities, the challenge in the implementation of multilayer optical networks lies in realizing multiple optical layers without resorting to electronic components. Here we present a novel framework that uses multiple scattering, and which is capable of synthesizing programmable linear and nonlinear transformations concurrently at low optical power by leveraging the nonlinear relationship between the scattering potential, represented by data, and the scattered field. Theoretical and experimental investigations show that repeating the data by multiple scattering enables nonlinear optical computing with low-power continuous-wave light. Moreover, we empirically find that scaling of this optical framework follows a power law.

## Abstract (web summary)

Multiple scattering capable of synthesizing programmable linear and nonlinear transformations concurrently at low optical power in the order of milliwatts continuous-wave power for optical computing is demonstrated, paving the way for ultra-efficient, low-power all-optical neural networks.

## Main

Optical computing has re-emerged as an alternative to electronics for performing computations and handling information, particularly in the context of artificial intelligence applications. Optical neural networks (ONNs) hold promise in terms of speed and energy efficiency compared with traditional electronic computing^1^. However, the development of fully optical ONNs has proven to be a challenging task due to the need for incorporating both linear and nonlinear computations within the optical domain^2^. Although several approaches have demonstrated efficient optical computing hardware for linear calculations^3–8^, effectively integrating these capabilities with nonlinear computations remains a substantial obstacle to the complete realization of ONNs. Researchers have explored nonlinear light–matter interactions in the context of reservoir computing^9–11^, employing high-intensity pulsed lasers for nonlinear data processing^12–15^. Additionally, the complex dynamics observed in multimode laser cavities interacting with external optical signals are employed for low-power nonlinear transformations in reservoir computing^16^. Platforms like the integrated meshes of Mach–Zehnder interferometers^3^, diffractive neural networks^6,17,18^, micro-ring resonators^7,19^ and free-space linear systems^5,8,20,21^ have facilitated linear calculations. However, nonlinear computations have relied on optoelectronic nonlinearity or electronic computation, resulting in limitations such as non-programmable optoelectronic nonlinearity and high energy consumption. Accordingly, there is a need to find a low-power flexible solution to implement programmable nonlinear operations in the optical domain to fully harness the low-power computing potential inherently offered by linear optics.

It has been shown previously that a purely linear transformation can be implemented with a stack of two-dimensional (2D) diffractive layers^22^. The Ozcan group has applied this approach to ONNs employing additive manufacturing techniques^6,18^. These deep learning-enabled multilayer diffractive processors enable computation by facilitating the propagation of free-space light through a sequence of structured passive scattering surfaces. This optical processing technique leverages the 3D connectivity between nodes in consecutive layers, achieved via diffraction, thereby providing a path to scalability^23^. However, one limitation of this approach is that the nonlinearity is limited to square-law detection at the output, which limits the realization of complex ONNs.

Another avenue that can be explored is the relationship between the scattering potential and the scattered light. At low intensity levels, the propagation of light through a scattering medium exhibits linearity in terms of the relation between the input and output light field, but the output light can have a nonlinear dependence on the data encoded in the scattering potential. This form of nonlinearity is referred to as structural nonlinearity, and it has been investigated by Eliezer and colleagues using multiple scatterings within an integrating sphere^24^.

In this Article we present a programmable framework called nonlinear processing with only linear optics (nPOLO) for the all-optical realization of neural networks using a low-power continuous-wave laser and diffractive layers. The nPOLO framework enables simultaneous linear and nonlinear operations within the optical domain. In this way, nPOLO unifies multilayer light modulation and structural nonlinearity such that the collective impact of data-modulated layers on propagating light generates a high-order nonlinear transformation of the data. The data are repetitively embedded into the modulation layers, combined with trainable parameters that enable the desired relationship (linear and nonlinear) between the data and the output field. Our results demonstrate that increasing the number of layers and data repetitions leads to the generation of higher-order nonlinearities, such as polynomial orders, which include cross-terms among the different elements of the input data. To illustrate the effectiveness of data repetition, we conducted a comparative analysis of the performance obtained between repeating the data in each modulation layer and presenting the data only once. Our results demonstrate that, when both systems have an equal number of degrees of freedom in terms of the displayed pixels in the modulation layers, the data repetition approach consistently achieves higher accuracy scores and exhibits improved robustness against experimental imperfections and simulated noise. Overall, our findings showcase the ability of the nPOLO framework to synthesize a learnable both linear and nonlinear data transform in a hybrid optical–digital neural network using only low-power continuous-wave light.

## nPOLO framework

**Fig. 1:**
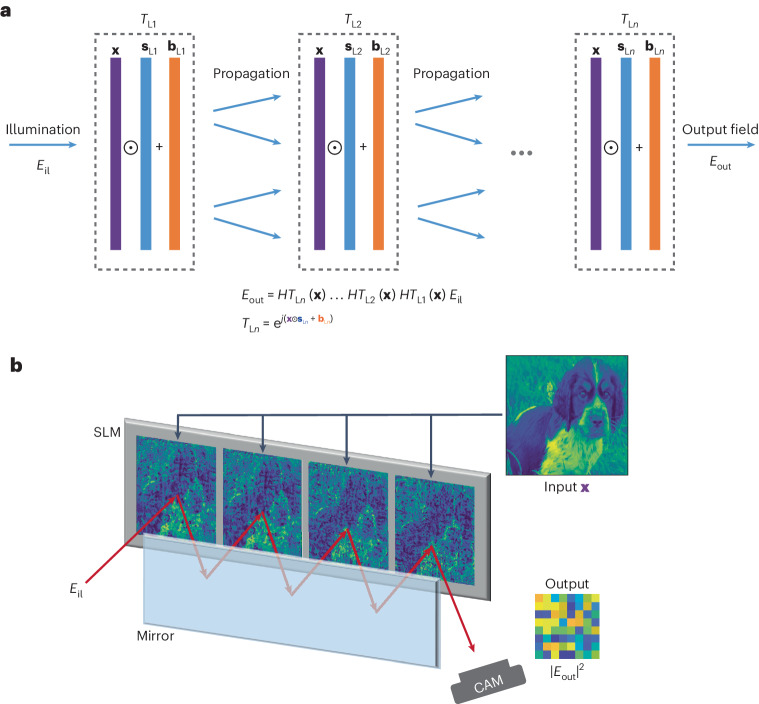
The nPOLO framework. **a**, Computation scheme, showing multiple modulation layers (*T*_L__*n*_) within the framework. The data (**x**) are presented on these layers, accompanied by trainable scaling (**s**_L__*n*_) and bias (**b**_L__*n*_) parameters to optimize the transformation. Propagation is represented by *H* (Toeplitz matrix). **b**, The physical implementation of nPOLO, featuring a single-pass multi-bounce cavity configuration. This implementation consists of a SLM and a mirror, positioned in a way that enables the realization of consecutive layers on the SLM side by side. The propagation distance is determined by the reflection from the mirror, allowing the light to propagate between the layers. Output light is captured with a camera. CAM, camera.

The core of the nPOLO technique involves utilizing multiple data planes that are evenly spaced. The physical implementation of nPOLO includes a liquid-crystal spatial light modulator (SLM) and a mirror positioned opposite it^25^, allowing the simultaneous display of multiple modulation planes on a single SLM device. This configuration forms a multi-bounce single-pass cavity, where each plane serves as a reflecting surface that modulates the phase of light as it propagates from one plane to the next. Figure 1 provides an unfolded representation of the multi-bounce nPOLO architecture. We can write the output field as a function of the modulation layers as follows:

1 $${E}_{\text{out}}={H}{T}_{\mathrm{L}n}({\bf{x}})\ldots {H}{T}_{\mathrm{L}2}({\bf{x}}){H}{T}_{\mathrm{L}1}({\bf{x}}){E}_{\rm{il}}$$

where *E*_il_ is the illumination beam, *T*_L__*n*_ is the transmittance of the *n*th modulation layer, and the modulation layer number goes up to *N*. *H* is the diffraction operator, which is the same for all the layers, as they are equally spaced. Without loss of generality, let us assume that we have an optical system that has an input aperture and output aperture that are sampled by a total number of *K* pixels. The input and output electric fields then become *K* × 1 vectors. We can write the incident field as a vector of ones, assuming the incident optical field is a plane wave propagating along the optical axis. Assuming the same number of pixels in the modulation layer as in the input/output apertures, *T*_L*n*_ becomes a diagonal matrix with size *K* × *K*. *H* is a *K* × *K* Toeplitz matrix that represents diffraction for an arbitrary distance^23^. If one directly inserts the data into *T*_L__*n*_, there is no trainable parameter to tune the transform. Thus, instead of direct use of the data in *T*_L__*n*_, we feed a linearly transformed version of it in the form of ${t}_{j}^{(n)}={s}_{j}^{(n)}{x}_{j}^{(n)}+{b}_{j}^{(n)}$ to introduce trainable parameters, where ${s}_{j}^{(n)}$ is a scaling parameter, ${x}_{j}^{(n)}$ is an element of the data vector, and ${b}_{j}^{(n)}$ is a bias parameter where *j* refers to indexing of the vectors and *n* is the layer index. When we expand equation (1) for the general case of complex modulation with these parameters, we obtain

2 $$\begin{array}{l}{o}_{i}=\mathop{\sum}\limits_{j=1}^{K}{h}_{{ij}}\left({s}_{j}^{(N)}{x}_{j}+{b}_{j}^{(N)}\right)\left(\ldots \mathop{\sum}\limits_{j=1}^{K}{h}_{{ij}}\left({s}_{j}^{(2)}{x}_{j}+{b}_{j}^{(2)}\right)\right.\\\left.\qquad\left(\mathop{\sum}\limits_{j=1}^{K}{h}_{{ij}}\left({s}_{j}^{(1)}{x}_{j}+{b}_{j}^{(1)}\right)\right)\ldots \right)=\mathop{\sum }\limits_{m=0}^{N}{\alpha }_{i,\,m}\left({\bf{s}},\,{\bf{b}}\right){{\bf{x}}}^{m}\end{array}$$

where *h*_*ij*_ is the element of the diffraction matrix. As seen in the left-hand side of equation (2), multiplication by **x** at each layer gives rise to polynomial orders. We can then write the overall transform in a generic polynomial form up to order *N* where coefficients (*α*_*i*__, *m*_) are functions of **s** and **b**, the right-hand side of equation (2).

Now, we look at how the input is transformed by the nonlinear activation function applied to the linear transformation of **x** for a perceptron in a digital neural network:

3 $${o}_{i}={g}\left(\,\mathop{\sum }\limits_{j=1}^{K}{w}_{{ij}}{x}_{j}+{b}_{i}\right)=\sum _{m}{\beta }_{i,\,m}\left({\bf{w}},\,{\bf{b}}\right){{\bf{x}}}^{m}$$

In equation (3), *w*_*ij*_ and *b*_*i*_ are the trainable weight and bias parameters of the perceptron, and *g* is an algorithmic nonlinear function of choice (sigmoid, ReLU and so on). The nonlinear activation function can be expressed as a polynomial expansion as well, where the coefficients (*β*_*i*__, *m*_) depend on **w** and **b**. In a multilayer perceptron (MLP), the output is a cascade of such polynomials, which is also a polynomial. Therefore, the functional form of equation (3) applies to an MLP as well. Equations (2) and (3) have a similar polynomial form except with different coefficients. Ideally, if the nPOLO and the MLP are both trained perfectly to implement the same function, then the coefficients of the two polynomial expansions should converge. In Supplementary Section 1 we present fully developed output vector **o** in terms of explicit polynomial orders.

Trainable parameters, in the form of scaling and bias, are applied to each pixel value of the data presented on the modulation layers. These parameters are trained digitally via a computer model (Methods). Once the desired nonlinear transformation is achieved on the computer, the parameters are applied to the multiple layers (adjacent planes on the SLM) in the experimental set-up, resulting in an intensity pattern that is recorded by a camera. Subsequently, a compact representation of the recorded camera pattern is obtained through average pooling, resulting in a 2D matrix of values such as a 4-by-4 or an 8-by-8 grid. This compact representation is then fed into a digital linear classifier, which processes the data via a single fully connected linear layer, thereby producing the final classification results.

## Results

**Fig. 2:**
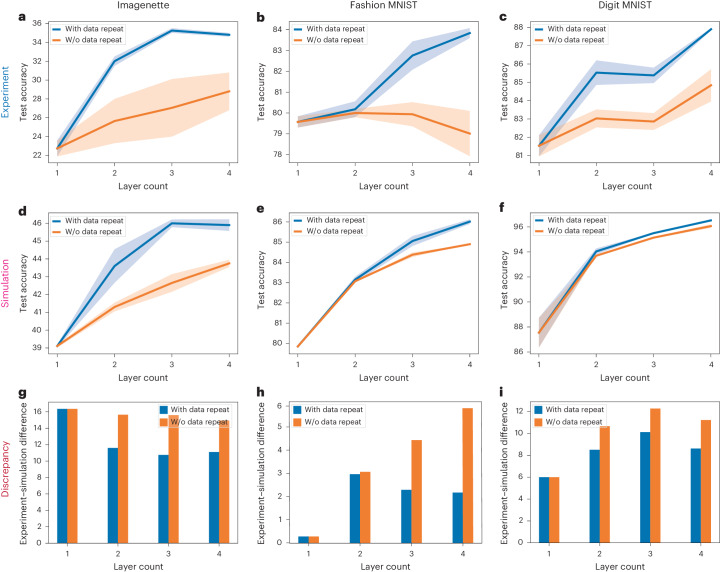
Classification accuracy results comparing two different schemes, one with data repeat and one without data repeat. **a**–**i**, Classification accuracy results obtained for the Imagenette (**a**,**d**,**g**), Fashion MNIST (**b**,**e**,**h**) and Digit MNIST (**c**,**f**,**i**) datasets, comparing two different schemes: one with data repeat and one without data repeat. The layer count (*N*) varies from 1 to 4 for both schemes. Each configuration is trained independently, resulting in layer masks that are applied to the SLM as phase masks. **a**–**c**, Experimentally obtained test accuracies for all datasets, representing the performance with and without structural nonlinearity. **d**–**f**, Test accuracies of the corresponding simulations, plotted for both schemes, as in experiments. The mean and s.d. values are obtained by testing the models trained from scratch. **g**–**i**, Mean accuracy difference between experimental and simulated results, shown as bar plots for varying layer number with and without data repeat. In plots **a**–**f**, solid lines are the mean results, and shaded regions represent ±1 s.d. Source data

By increasing the number of layers—that is, adjacent planes on the SLM—one can assess the impact of the polynomial orders resulting from structural nonlinearity. However, the increase in the number of layers also leads to an increase in the system’s degrees of freedom and space–bandwidth product. We thus devised an alternative comparison experiment to mitigate these effects. In our experiments, we maintained the same number of layers and pixels but modified the data allocation. Specifically, we initially incorporated the data only in the first layer, and the subsequent layers consisted exclusively of trainable bias parameters without any data or scaling parameters. To evaluate the performance of the nPOLO framework, we conducted experiments using the Imagenette, Fashion MNIST and Digit MNIST datasets^26–28^. The obtained numerical and experimental results are provided in Fig. 2.

We used 300 × 300 pixels on the SLM for each modulation layer. The original Fashion and Digit MNIST dataset samples contain 28 × 28-pixel images and the Imagenette dataset samples 320 × 320-pixel images. For the Fashion and Digit MNIST datasets we used 4 × 4 superpixels on the SLM, yielding a 75 × 75 grid for assigning trainable parameters, yielding 11,250 parameters (scaling and bias) per layer. We linearly upsampled the images of those datasets to 75 × 75. For the Imagenette dataset we linearly downsampled the images to 300 × 300 and used all the pixels for assigning trainable parameters, yielding 180,000 parameters (scaling and bias) per layer.

**Fig. 3:**
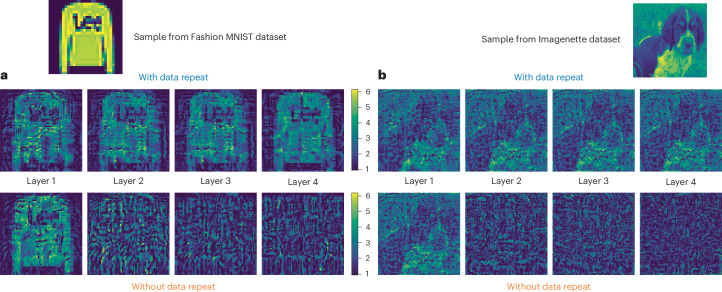
Samples of trained layer masks to be displayed on SLM for Fashion MNIST and Imagenette. **a**, Example sample from the Fashion MNIST dataset alongside its corresponding layer masks, combined with trainable parameters, according to both schemas. The colour code represents phase modulation ranging from 0 to 2π. **b**, Example sample from the Imagenette dataset, and its corresponding layer masks combined with trainable parameters for both schemas.

For clarity, we provide examples of the displayed masks in Fig. 3 in ‘with data repeat’ and ‘without data repeat’ configurations using an example from the Fashion MNIST and Imagenette datasets with four modulation layers. Supplementary Section 2 presents a comparative depiction of parameter allocation. The trainable parameters were optimized by computer simulation, where the physical light propagation was modelled using the beam propagation method (BPM).

**Fig. 4:**
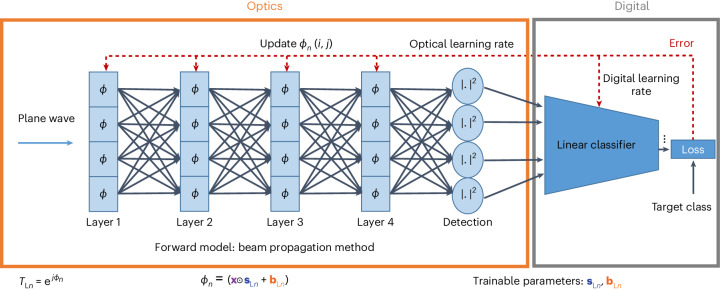
Co-training of optical trainable parameters and digital trainable parameters. Simulation model of the four-layer system, built using the beam propagation method, where layers are composed of pixels having phase *ϕ*, defined by trainable parameters (**s**_L__*n*_ and **b**_L__*n*_). Training of the optical layers and linear classifier is performed simultaneously using separate learning rates.

Because BPM consists of differentiable calculation steps, the error can be backpropagated to the trainable parameters, and they are optimized using stochastic gradient descent. In Fig. 4 we present the training scheme used, in which the digital model of the optical system and the digital classifier were co-trained for the classification tasks. By following this co-training approach, we obtained scaling and bias masks for different layer configurations, ranging from layer *N* = 1 to layer *N* = 4, both with and without data repetition. It is important to note that in the case of a single layer (*N* = 1), both data repetition options are equivalent, as we only had a single layer available to introduce the data.

Our experimental findings in Fig. 2a–c consistently demonstrate that when data are repeated across multiple layers, we achieve higher classification accuracy compared with configurations without data repetition. Moreover, increasing the number of layers also contributes to improved accuracy. We also observed that when the number of layers is held constant, eliminating data repetition leads to a reduction in accuracy. These results highlight the contribution of higher-order optical nonlinearities generated via data repetition. We observed a similar trend in our simulations while calculating the trainable parameters (Fig. 2d–f). Both experimental and simulated results validate the contribution of the nPOLO framework. However, the experimental accuracies were lower than the simulated ones. Figure 2g–i illustrates the accuracy difference between simulations and experiments in all three datasets. Interestingly, the decrease in accuracy is less pronounced in cases involving data repetition. We attribute this discrepancy to imperfections between the simulated model and the physical implementation, primarily the non-ideal phase response and flickering of the SLM. For example, we were able to reduce the discrepancy between the optical experiment and the digital simulation to 2% by upgrading the SLM for the Imagenette database with the data repeat configuration (Supplementary Section 3). Such engineering aspects of the nPOLO framework will be further explored in a follow-up study. We also trained a simple convolutional neural network to assess how nPOLO compared with a fully digital counterpart for these tasks and obtained comparable performance (Supplementary Section 4).

### Scaling study

**Fig. 5:**
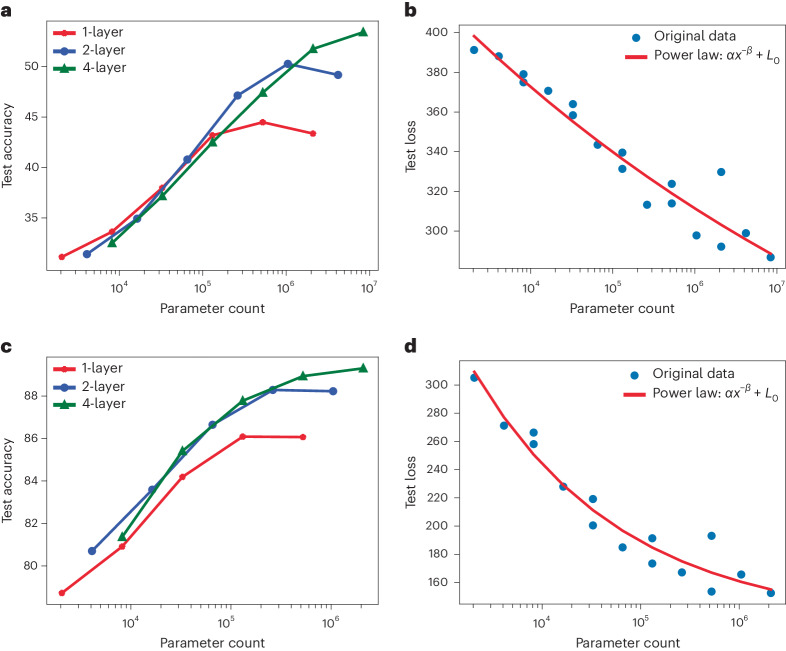
Scaling dynamics of nPOLO with increasing parameter count, showing test accuracies and losses for both the Imagenette and Fashion MNIST datasets across various parameter configurations. Manipulation of layer width and depth enables the realization of different parameter counts. **a**–**d**, Detailed breakdown of test accuracies for different layer counts (1, 2 and 4) individually (**a**,**c**) and all test results in relation to parameter count, irrespective of the specific layer number (**b**,**d**) for the Imagenette (**a**,**b**) and Fashion MNIST (**c**,**d**) datasets. Results (**b**,**d**) are fitted to a power function defined by parameters *α*, *β* and *L*_0_, where *x* represents parameter count. Source data

The performance is influenced by three factors: the number of trainable parameters, the dataset size and the available compute budget for training^29^. Although the dataset size remains constant in our study, the model size is subject to variation within the constraints of a limited computational budget. Specifically, we explored the impact of changing the model size on the performance of nPOLO, determined by the width, *W*^2^, (the number of SLM pixels in the illuminated 2D patch) of its diffractive layers and the depth, *N*, denoting the number of diffractive layers. Our empirical investigation involved varying *W* and *N* for classification tasks of the Imagenette and Fashion MNIST datasets (Methods). The findings are summarized in Fig. 5. The parameter count of our model is 2*NW*^2^. The factor of 2 is included because of the two trainable parameters per pixel. Initially, we observe a continuous upward trend in performance as the parameter count increases, regardless of *N* and *W*. In other words, configurations with different depths perform similarly for the same parameter count, up to a few hundred thousand, as shown in Fig. 5a,c. However, further increase in the parameter count leads to performance saturation. This phenomenon is also observed in conventional digital neural networks. Deeper implementations of nPOLO (*N* > 1) extend the onset of this saturation point. Recent studies at OpenAI have revealed that the scaling of deep MLP networks adheres to a power-law relationship between the test loss and the size of the model^29,30^, extending across numerous layers and billions of parameters. The test loss of the simulated nPOLO is plotted in Fig. 5b,d against the number of parameter counts. This reveals a power-law scaling within the examined network depth, encompassing up to four layers and ~8.4 million parameters. Despite the orders of difference in parameter counts, the presence of power-law scaling in this range is encouraging for the development of future large-scale networks.

## Discussion

We introduced trainable scaling and bias parameters for the pixel values of the data displayed on the SLM to synthesize a programmable computation. We observed that the contribution of structural nonlinearity became more pronounced when dealing with more challenging classification tasks. The benefit of using repeated data versus not using repeated data was more pronounced for Imagenette than for Digit MNIST. This disparity arises because Digit MNIST represents an easier task, and the structural nonlinearity becomes redundant in the presence of the square-law detection nonlinearity.

The scaling study we performed revealed that as the model size of nPOLO increases the performance improves, and the optimal performance relies on a balanced scaling of width and depth of the model. Remarkably, similar properties are also found in digital deep neural networks^30,31^. Future studies could explore alternative approaches of employing trainable parameters or implementing different functional forms, such as using convolutional kernels or eigenmodes of the optical system as trainable parameters, similar to investigations conducted for fibre-based optical-learning machines^32^. During the final stage of this work, we became aware of an independent and different approach to perform passive optical nonlinearity, exploiting reflections inside a disordered cavity^33^. More recently, another article appeared investigating, through digital only simulations, structural nonlinearity for the implementation of neural networks^34^.

An interesting observation is the increased robustness of data repetition across multiple layers against experimental imperfections. We initially noticed this phenomenon during experiments conducted on different datasets and layer configurations. To further investigate this, we introduced phase noise and misalignment in BPM simulations to emulate experimental imperfections while keeping the trained masks fixed (Supplementary Section 3). Gradually increasing the simulated noise level or misalignment degree, we observed that the configurations with data repetition exhibited greater robustness. The configuration without data repetition experienced a more rapid drop in accuracy, consistent with the experimental results. This finding strengthens the argument for the noise robustness of the data repetition scheme, as we have empirical data from both experiments and simulations. One possible explanation for this phenomenon is that by introducing the data multiple times, the model learns multiple paths from the input data to the output plane during training, resulting in not only higher polynomial orders but also cross-terms that couple with different optical paths and reach the detector plane. The existence of multiple routes for highlighting useful features in the output plane may make the data repetition scheme less susceptible to noise.

Our scaling study demonstrates a power-law scaling trend akin to the observations in OpenAI’s deep neural network studies^29,30^. While exploring deeper models with more than four layers for improved performance, we found that increased depth did not yield substantial enhancements. We hit the dataset bottleneck, as we observe overfitting for the large number of parameters that deeper models employ. The increased deviations towards a high parameter count (Fig. 5) also exist for this reason. This aligns with the findings reported in refs. ^29,30^, whereby empirical performance individually exhibits a power-law relationship with three factors—model size, dataset and compute budget—when not constrained by the other two. In practical terms, pursuing deeper models might offer diminishing returns unless accompanied by adjustments in dataset size and the employment of greater computational resources. We note that the highest parameter count in the scaling study yielded ~4.2 million pixels for Imagenette and ~1 million for the Fashion and Digit MNIST datasets, which can be employed using commercially available devices. Other possibilities to further scale the number of parameters include using multiple SLMs or other structured media such as computer-generated holograms^35^ or volume holograms^36,37^, instead of the flat mirror, to accommodate additional parameters to boost performance.

Overall, the nPOLO framework presents a novel approach for generating optical nonlinearity using low-power optical devices, eliminating the need for electronic components to achieve higher orders of nonlinearity. Networks implemented with structural nonlinearities are not MLPs, but they can be trained to reach comparable performance and they have similar scaling laws. We also discovered that the introduction of data repetition to generate polynomial nonlinearities enhances robustness against noise. Note that this framework is applicable to the cascade of any optical linear system, including integrated-waveguide Mach–Zehnder interferometers^3^. These characteristics make nPOLO a promising platform for realizing ONNs.

## Methods

### Digital training

Optimization methods have already been demonstrated to reconstruct 3D phase objects from experimental recordings of 2D projections^38,39^. In ref. ^38^, the forward model in the optimization is the BPM. The iterative error reduction scheme and multilayer structure of the BPM resembles a multilayer neural network. Accordingly, this method is referred to as ‘learning tomography’ (LT). We show that, instead of imaging an object, we can reconstruct the 3D structure that performs the desired task as defined by its input–output functionality^35^. To establish the target functionality, 3D phase modulation (either through a continuous medium or multiple planes) and the scattered field caused by the phase modulation must be modelled accurately. Unlike conventional reconstruction algorithms that rely on first-order approximations, LT incorporates higher-order scattering effects by employing BPM. The LT algorithm involves an iterative reconstruction process using the forward model, along with the constraints arising from experimental considerations such as the pixel pitch of the SLM. In this Article we have adapted this approach, presented in ref. ^35^ (where additional details can be found), to generate scaling and bias parameters for the demonstrated classification tasks. The output intensity pattern of the forward model is average pooled to yield a 4 × 4 matrix for each sample of the Fashion MNIST dataset and an 8 × 8 matrix for each sample of the Imagenette dataset. These matrices are flattened to act as an input layer of a digital classifier that has ten output neurons for each class of the datasets, without any hidden layer and nonlinear activation function. The trainable parameters employed in the BPM model and digital classifier are co-trained by a continuous error backpropagation where different learning rates are assigned to digital weights (10^−4^) and optical scaling and bias parameters (10^−3^), using categorical cross-entropy as the loss function. We used batch learning with a batch size of 20 and a random shuffle in every batch. PyTorch libraries were used for the whole training process.

For the scaling study, we followed an approach akin to classical neural networks by augmenting the number of trainable parameters in nPOLO. The parameter count per plane in nPOLO is *W*^2^, where *W* is the plane’s width. We conducted a sweep of *W* values from 32 to 1,024 (up to 512 for Fashion MNIST) for bounce numbers of 1, 2 and 4. The test accuracies over 50 epochs for the Imagenette and Fashion MNIST datasets are presented in Fig. 5. During scale-up studies we encountered overfitting, and slight data augmentation (random flip and rotation) was introduced to reduce this.

### Experimental set-up

A photograph of our optical set-up is presented in Supplementary Section 3. In our experiments, we employed a continuous-wave Solstis M2 laser operating at a wavelength of *λ* = 850 nm. The mirror we used had a width of 11 mm, providing ample space for the four reflections. To deliver the beam to the SLM, we implemented 4*f* imaging to relay the beam reflected from a digital micromirror device (DMD). The use of a DMD offered the advantage of flexible beam sizing. Specifically, we configured the beam shape as a square with a side length of 2.4 mm, corresponding to a 300 × 300-pixel area on the SLM. It is worth noting that the SLM utilized in our set-up has a pixel pitch of *Λ* = 8 μm. To construct a multi-bounce cavity, we utilized a Holoeye Pluto SLM and positioned the mirror at a distance of *d* = 15.2 mm from the SLM screen. This distance was set so that diffraction from one corner pixel of a layer can reach the opposite corner of the subsequent layer (*d* × *λ*/*Λ* ≥ 2.4 mm). The distance between the SLM and mirror enabled all-to-all pixel connectivity. Zero-order reflection from the SLM was used, which has 69% percent reflectivity. This configuration allowed the input beam to undergo four reflections from the mirror. Therefore, the total transmission due to the SLM efficiency after four bounces was 23%. After the fourth bounce, the beam on the SLM was magnified by a factor of 1.2, and the resulting output intensity was detected by a complementary metal–oxide–semiconductor (CMOS) camera with a pixel pitch of 3.45 µm. The corresponding beam in the camera occupied an area of 834 × 834 pixels. During image acquisition, we applied average pooling to resize the obtained images to either 4 × 4 or 8 × 8 dimensions. For the Imagenette dataset, we used the entire training and test samples as originally prepared. For the Digit and Fashion MNIST datasets, we used the entire training set (60,000 samples) for the simulations, but we used the first 10,000 samples for the re-training of digital weights after the experiments, and we used the first 2,500 samples of the test set for blind testing of the experimental results.

## Online content

Any methods, additional references, Nature Portfolio reporting summaries, source data, extended data, supplementary information, acknowledgements, peer review information; details of author contributions and competing interests; and statements of data and code availability are available at 10.1038/s41566-024-01494-z.

## Supplementary information

Supplementary Information Supplementary Figs. 1–7, Discussion 1–4 and Tables 1 and 2.

## Source data

Source Data Fig. 2 Data txt files of Fig. 2.

Source Data Fig. 5 Data txt files of Fig. 5.

## Data Availability

The datasets containing the raw information for the Imagenette dataset are from https://github.com/fastai/imagenette. The Digit and Fashion MNIST datasets can be retrieved from https://pytorch.org/vision/stable/datasets.html. Source data are provided with this paper.

## Code availability

All relevant code is available from the corresponding authors upon reasonable request.
