# Supplementary material for: Nonlinear processing with linear optics
Source: Nat Photonics. 2024 Jul 31;18(10):1076–82. doi: 10.1038/s41566-024-01494-z (PMC11449797; doi:10.1038/s41566-024-01494-z)
Supplement: Supplementary file 1 — Supplementary Figs. 1–7, Discussion 1–4 and Tables 1 and 2. [file 41566_2024_1494_MOESM1_ESM.pdf]

nature photonics

Supplementary information https://doi.org/10.1038/s41566-024-01494-z

Nonlinear processing with linear optics

In the format provided by the authors and unedited

Contents

Contents 1
Supplementary Material Section 1: Derivation of nonlinear response for 2-pixel layers 2
Supplementary Material Section 2: Allocation of trainable parameters 5
Supplementary Material Section 3: Experimental Setup and noise robustness 6
    Numerical study to probe noise robustness 6
    Closing the discrepancy gap 8
Supplementary Material Section 4: Comparison with fully digital networks 9
Supplementary Reference 10

Supplementary Material Section 1: Derivation of nonlinear response for 2-pixel layers

We present an analysis of a simplified optical system where each plane/layer consists of two pixels (see Supplementary Fig. 1) and a propagation step whose system response is expressed by a matrix with linear coefficients. The generalization of this analysis to an arbitrary number of pixels in each layer is straightforward. We provide the investigation for Modulation layer number N=1, N=2, and N=3 for ease of explanation and the conclusions are valid for arbitrary N by inductive reasoning.

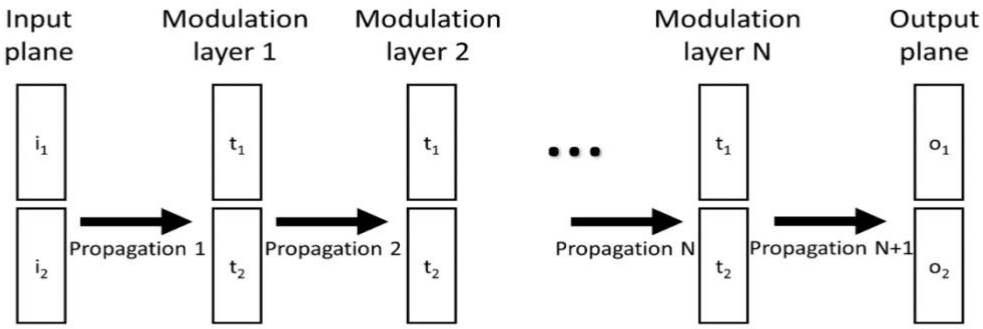

Supplementary Figure 1 *shows a sketch of the simplified optical system where each plane/layer consists of two pixels.*

The system response for Modulation layer N=1 is the following:

$$\boldsymbol{o} = \boldsymbol{P_2 T P_1 i} \tag{S.1}$$

Where *o* is the output vector (Electric field), *i* is the input vector (Electric field), P1 is the propagation matrix 1, P2 is the propagation matrix 2, and T is the modulation matrix. For P1, we assume zero propagation for simplicity without losing generality and for P2 we use a Toeplitz matrix to represent diffraction for an arbitrary distance. For a two-pixel per layer system we simply have the following:

$$\boldsymbol{P_1} = \begin{bmatrix} 1 & 0 \\ 0 & 1 \end{bmatrix}$$

$$\boldsymbol{P_2} = \begin{bmatrix} h_{11} & h_{12} \\ h_{21} & h_{22} \end{bmatrix} \tag{S.2}$$

$$\boldsymbol{T} = \begin{bmatrix} t_1 & 0 \\ 0 & t_2 \end{bmatrix}$$

Hence:

$$\begin{bmatrix} o_1 \\ o_2 \end{bmatrix} = \begin{bmatrix} h_{11} & h_{12} \\ h_{21} & h_{22} \end{bmatrix} \begin{bmatrix} t_1 & 0 \\ 0 & t_2 \end{bmatrix} \begin{bmatrix} i_1 \\ i_2 \end{bmatrix} \tag{S.3}$$

And we have:

$$\begin{bmatrix} o_1 \\ o_2 \end{bmatrix} = \begin{bmatrix} h_{11}t_1 i_1 + h_{12}t_2 i_2 \\ h_{21}t_1 i_1 + h_{22}t_2 i_2 \end{bmatrix} \tag{S.4}$$

Following similar steps for Modulation layer N=2 (assuming P3=P2 without loss of generality). Note that the data in layer N=2 is the same as layer N=1.

$$\begin{bmatrix} o_1 \\ o_2 \end{bmatrix} = \begin{bmatrix} h_{11} & h_{12} \\ h_{21} & h_{22} \end{bmatrix} \begin{bmatrix} t_1 & 0 \\ 0 & t_2 \end{bmatrix} \begin{bmatrix} h_{11}t_1 i_1 + h_{12}t_2 i_2 \\ h_{21}t_1 i_1 + h_{22}t_2 i_2 \end{bmatrix}$$

$$\begin{bmatrix} o_1 \\ o_2 \end{bmatrix} = \begin{bmatrix} h_{11}^2 t_1^2 i_1 + h_{11}h_{12}t_2 t_1 i_2 + h_{12}h_{21}t_1 t_2 i_1 + h_{12}h_{22}t_2^2 i_2 \\ h_{21}h_{11}t_1^2 i_1 + h_{21}h_{12}t_2 t_1 i_2 + h_{22}h_{21}t_1 t_2 i_1 + h_{22}^2 t_2^2 i_2 \end{bmatrix} \tag{S.5}$$

Hence, we reach a nonlinear relationship between the output field o_1, o_2 and the data plane t_1, t_2. When intensity detection is employed as the acquisition method, the obtained output will be the absolute square of the field, providing a 4th order polynomial of the parameters inserted in the modulation layers for the specific case of 2 layers (N=2). Clearly, when data is introduced in the modulation layers, we obtain a nonlinear processing of the data at the output plane either by detecting the field (by a holographic recording) and/or the intensity (by a simple detector, which can be CMOS, CCD, etc.). The input field *i* can be a programming parameter to change the effective transform. For simplicity, we will continue with a plane wave input without loss of generality:

$$\begin{bmatrix} i_1 \\ i_2 \end{bmatrix} = \begin{bmatrix} 1 \\ 1 \end{bmatrix} \tag{S.6}$$

For a phase-only modulation, we have the following relation for the modulation terms:

$$t_i = e^{j\phi_i} \text{ where } j = \sqrt{-1}. \tag{S.7}$$

In this case, the data becomes ϕ_i. Then we have:

$$\begin{bmatrix} o_1 \\ o_2 \end{bmatrix} = \begin{bmatrix} h_{11}^2 e^{j2\phi_1} + h_{11}h_{12}e^{j(\phi_1+\phi_2)} + h_{12}h_{21}e^{j(\phi_1+\phi_2)} + h_{12}h_{22}e^{j2\phi_2} \\ h_{21}h_{11}e^{j2\phi_1} + h_{21}h_{12}e^{j(\phi_1+\phi_2)} + h_{22}h_{21}e^{j(\phi_1+\phi_2)} + h_{22}^2 e^{j2\phi_2} \end{bmatrix} \tag{S.8}$$

In the above expression, there is no polynomial order of the data at the output electric field, although the relation is nonlinear due to the summation of exponentials. When we detect the intensity, we obtain the following:

$$\begin{bmatrix} I_1 \\ I_2 \end{bmatrix} = \begin{bmatrix} |o_1|^2 \\ |o_2|^2 \end{bmatrix} = \begin{bmatrix} \left| h_{11}^2 e^{j2\phi_1} + h_{11}h_{12}e^{j(\phi_1+\phi_2)} + h_{12}h_{21}e^{j(\phi_1+\phi_2)} + h_{12}h_{22}e^{j2\phi_2} \right|^2 \\ \left| h_{21}h_{11}e^{j2\phi_1} + h_{21}h_{12}e^{j(\phi_1+\phi_2)} + h_{22}h_{21}e^{j(\phi_1+\phi_2)} + h_{22}^2 e^{j2\phi_2} \right|^2 \end{bmatrix}$$

$$= \begin{bmatrix} DC + C_1\cos(\phi_1 - \phi_2 + \theta_1) + C_2\cos(2(\phi_1 - \phi_2 + \theta_2)) \\ DC + C_3\cos(\phi_1 - \phi_2 + \theta_3) + C_4\cos(2(\phi_1 - \phi_2 + \theta_4)) \end{bmatrix} \quad \text{(S.9)}$$

In the above expression, the DC term refers to the grouping of the terms that do not depend on the SLM phase pattern ϕ_i.The constant terms C_i represent the electric field amplitude resulting from light propagation between layers. θ_i is the additional phase bias of the constants arriving from propagation matrix. The elements of the propagation matrix are complex valued. Note that the intensity detection yields cosine terms. The integer multiplier (the factor 2) in the second cosine term comes from the fact that there are two modulation layers. Note that this term has polynomial orders:

$$\cos(2\theta) = 2\cos^2(\theta) - 1 \quad \text{(S.10)}$$

Hence, intensity detection provides the nonlinearity (cosine) and where the multiple modulation layers provide the polynomial orders of the cosine term. Similarly, when adding a third modulation layer N=3, we have:

$$\begin{bmatrix} o_1 \\ o_2 \end{bmatrix} = \begin{bmatrix} h_{11}^3 t_1^3 + \left(h_{11}^2 h_{12} + 2h_{12}h_{21}h_{11}\right) t_2 t_1^2 + \left(h_{11}h_{12}h_{22} + h_{12}^2 h_{21} + h_{12}h_{21}h_{22}\right) t_1 t_2^2 + h_{12}h_{22}^2 t_2^3 \\ h_{21}h_{11}^2 t_1^3 + \left(h_{21}h_{11}h_{12} + h_{21}^2 h_{12} + h_{11}h_{21}h_{22}\right) t_2 t_1^2 + \left(h_{22}^2 h_{21} + 2h_{12}h_{21}h_{22}\right) t_1 t_2^2 + h_{22}^3 t_2^3 \end{bmatrix}$$

$$\begin{bmatrix} I_1 \\ I_2 \end{bmatrix} = \begin{bmatrix} DC + C_1\cos(\phi_1 - \phi_2 + \theta_1) + C_2\cos(2(\phi_1 - \phi_2 + \theta_2)) + C_3\cos(3(\phi_1 - \phi_2 + \theta_3)) \\ DC + C_4\cos(\phi_1 - \phi_2 + \theta_4) + C_5\cos(2(\phi_1 - \phi_2 + \theta_5)) + C_6\cos(3(\phi_1 - \phi_2 + \theta_6)) \end{bmatrix} \quad \text{(S.11)}$$

Noting that:

$$\cos(3\theta) = 4\cos^3(\theta) - 3\cos(\theta) \quad \text{(S.12)}$$

By induction, it is obvious that the polynomial orders increase with the number of modulation layers N. In summary, complex modulation (amplitude and/or phase) or with only amplitude modulation in the different layers yield a nonlinear relationship between the output field and the modulation parameters. In our implementation, intensity detection is performed along with phase only modulation. This also results in nonlinear relationship via polynomial orders of sinusoidal terms induced by intensity detection. Essentially, the trainable parameters S_i, B_i that are added to each pixel of the data layers change the evaluation regime on the cosine polynomials. Hence, their effect is analogous to the nonlinear activation in deep neural networks.

Supplementary Material Section 2: Allocation of trainable parameters

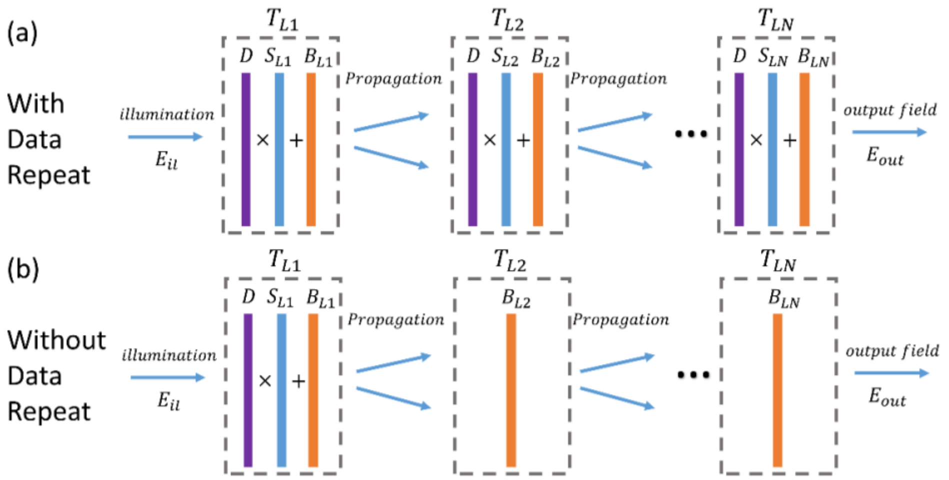

Supplementary Figure 2. Comparison scheme to observe the effect of structural nonlinearity with equivalent systems in terms of space-bandwidth product. *a) Cascaded modulation layers where each layer comprises the input data as given in Figure 1a as well. b) Cascaded modulation layers where only the first layer comprises the input data and the consecutive layers contain trainable bias parameters.*

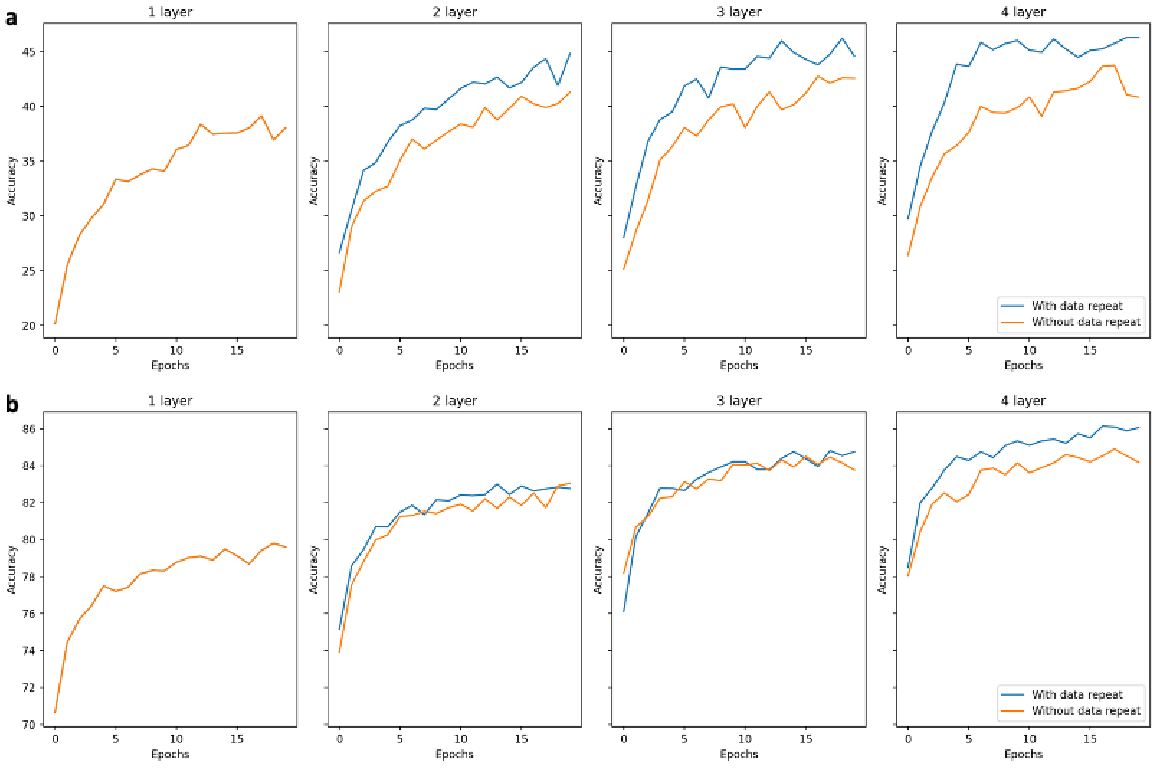

Supplementary Figure 3 demonstrates the test accuracy results obtained during training of the Imagenette (a) and Fashion MNIST (b) datasets on two different schemas: one with "data repeat" and one without. *Note that, one layer corresponds to the same configuration for both schemas. Each configuration is trained for 20 epochs.*

Supplementary Material Section 3: Experimental Setup and noise robustness

The results presented in Fig. 2 of the main text are obtained using the experimental setup depicted below.

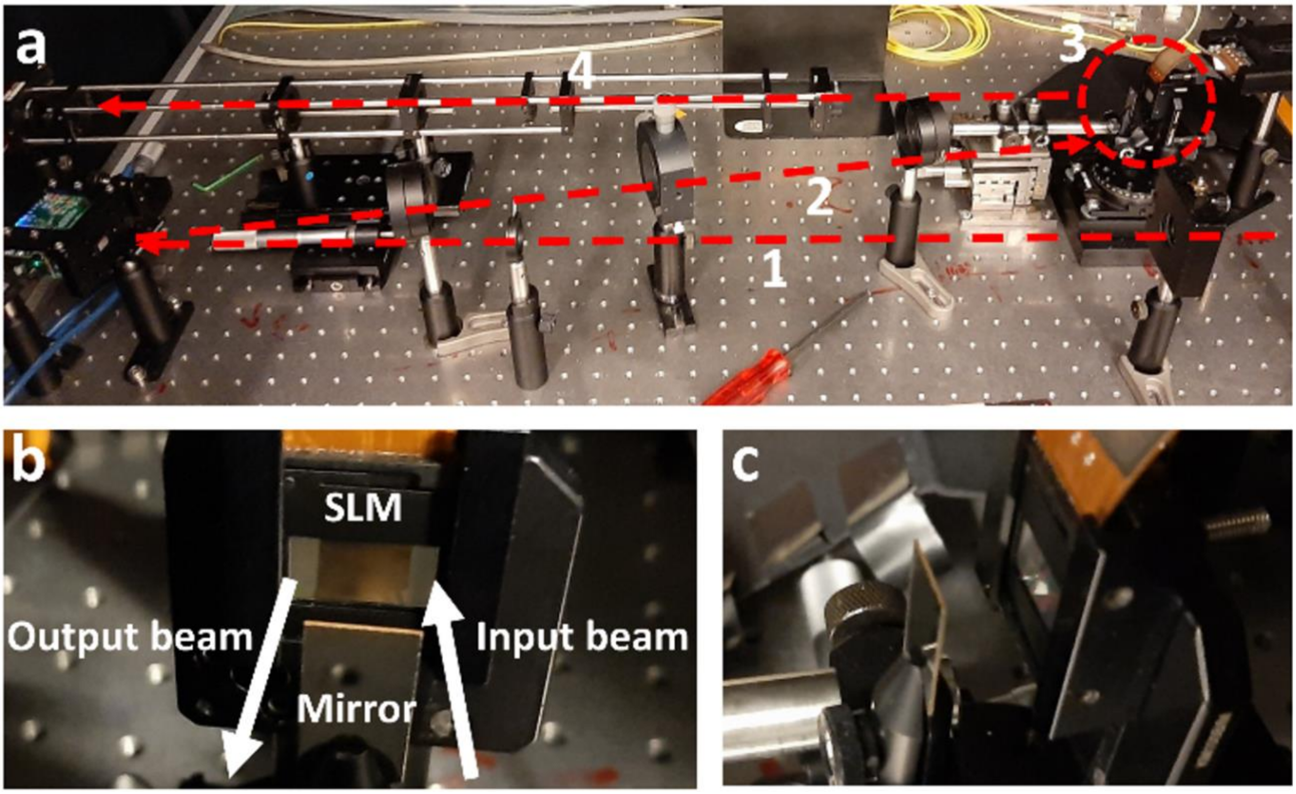

Supplementary Figure 4 demonstrates the experimental setup. *a) The experimental setup. 1-collimated laser beam path, 2-a Digital Micromirror Device (DMD) emulates input aperture and a 4F imaging system relays the light into multi-bounce cavity, 3-multi-bounce cavity, 4-another 4F imaging system relays the output of the multi-bounce cavity on a camera. b) Front-view of the multi-bounce cavity in the setup c) side-view of the multi-bounce cavity.*

Numerical study to probe noise robustness

To investigate the influence of phase noise on SLM (Spatial Light Modulator), we conducted simulations by introducing random additive phase noise. We applied the forward model to all three datasets for various the levels of phase noise. The noise was randomly generated from a normal distribution and applied to every pixel of the four layers. Subsequently, we determined the output accuracy based on the simulated outputs affected by the introduced noise. Supplementary Figure 4 demonstrates that as the strength of the noise increases, the accuracy drop in “without data repetition” occurs more rapidly. This numerical analysis corroborates the findings from our experimental observations.

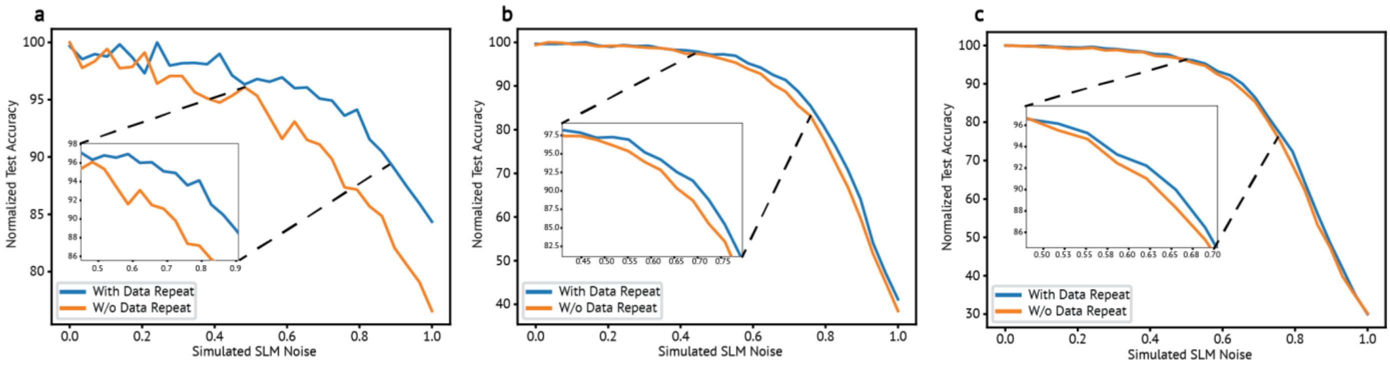

Supplementary Figure 5 illustrates the effect of phase noise on pixels. *The plots depict the test accuracy drop as the phase noise on the Spatial Light Modulator (SLM) is incrementally increased from zero to one (corresponding to 2π), both for the cases with data repetition and without data repetition. The obtained test accuracies are normalized with respect to the scenario where the phase noise is zero so that accuracies start from 100%. Panels a, b, and c correspond to the datasets: Imagenette, Fashion MNIST, and Digit MNIST, respectively. The insets provide a zoomed view of the difference between the cases with data repetition and without data repetition.*

To probe the influence of misalignment of the illumination beam and the mirror in terms of angular mismatches, we trained three models where the training included random XYZ shifts of the layers and the 5% phase noise in SLM display. We used the three models for inference by first varying the mirror angle and keeping the illumination angle fixed at the correct value. For each inference we re-trained the digital classifier weights and recorded the test accuracies. The obtained plots with and without data repeat configurations are presented in Supplementary Figure 6a-c where the highest accuracies are normalized to 100% to observe the different trends with and without data repeat. We clearly see that the data repeat configuration is more robust to misalignment and the difference gets more pronounced as the difficulty of the task increases. The input angle varies from -1 to 1 milliradians in both X and Y directions at the same time. Hence, for the X-axes of the plots, we multiply this range with $\sqrt{2}$ to present the angular mismatch in y=x line. We re-trained the digital classifier weights for each angle. That is why there are small oscillations on top of the general trend due to re-training of digital weights. If we keep the digital classifier weights fixed, the accuracy values quickly approach to random guess with misalignment. The same numeric experiments are conducted with the mirror misalignment angle (with respect to SLM display) as presented in Supplementary Figure 6d-f. Since the accuracy dependence is observed to be higher for this case, the angular range is decreased to half, i.e. -0.5 to 0.5 milliradians in both X and Y directions at the same time.

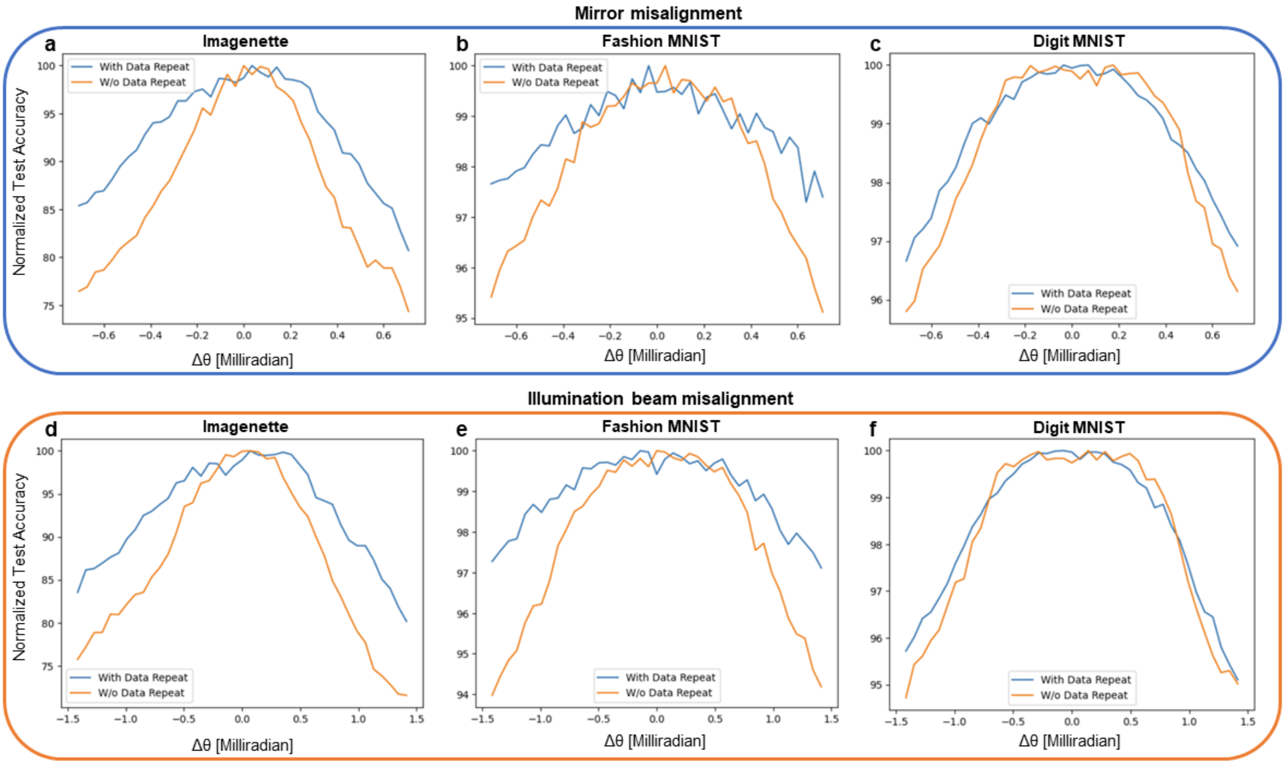

Supplementary Figure 6 illustrates the effect of angular misalignment of the illumination beam and the mirror. *The plots depict the test accuracy drop as the angular misalignment is introduced, both for the cases with data repetition and without data repetition. The obtained test accuracies are normalized so that accuracies start from 100% for both data repeat and without data repeat. Panels a, b, and c correspond to the datasets: Imagenette, Fashion MNIST, and Digit MNIST, respectively for the illumination angle misalignment numerical experiment. Panels d, e, and f correspond to the datasets: Imagenette, Fashion MNIST, and Digit MNIST, respectively for the mirror angle misalignment numerical experiment.*

Closing the discrepancy gap

To understand the discrepancy between the numerical investigations and the experiment, we replaced the SLM with a Meadowlark SLM that has less flickering. In addition, we designed, and 3D printed a mirror holder to minimize the misalignment between the SLM display and the mirror. The holder is attached to the SLM display to prevent relative drift of the display and the mirror. Since the new SLM has a different pixel pitch (9.2 µm instead of 8 µm), we placed the mirror and the SLM further apart (17.1 mm instead of 15.2 mm) to keep the level of connectivity by diffraction at the same order. Moreover, since the illumination beam is kept the same, we had to reduce the number of employed pixels in a single 2D patch to 260-by-260 instead of 300-by-300. The new multi-bounce cavity is shown in Supplementary Figure 7. We re-trained a model considering these adjustments for a 4-layer system with data repeat and without data repeat configurations for the classification task of the Imagenette dataset. The results are presented in Supplementary Table 1. Because of the reduced number of pixels (hence the trainable parameters) and the increased noisy conditions during in-silico training to obtain more robust masks against experimental imperfections, the accuracy values obtained in simulations are lower whereas the experimental accuracy value for the data repeat configuration increased. We will

further explore the underlying phenomena on robustness of nPOLO framework when it is trained under noisy conditions in the future.

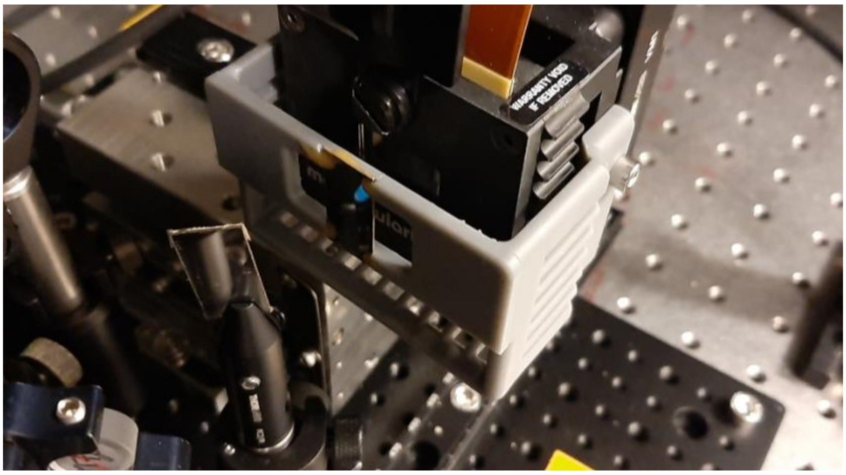

Supplementary Figure 7 illustrates the upgraded setup with a new SLM display and mirror holder.

Supplementary Table 1. *The accuracy values obtained with the new nPOLO setup for Imagenette dataset using 4 layers.*

<table>
<tr><td rowspan="2">With Data repeat</td><td>Simulation test accuracy: 42.88%</td><td rowspan="2">Discrepancy: 1.78%</td></tr>
<tr><td>Experiment test accuracy: 41.10%</td></tr>
<tr><td rowspan="2">Without Data repeat</td><td>Simulation test accuracy: 33.86%</td><td rowspan="2">Discrepancy: 6.60%</td></tr>
<tr><td>Experiment test accuracy: 27.26%</td></tr>
</table>

Supplementary Material Section 4: Comparison with fully digital networks

We trained convolutional neural networks (CNN) that can provide close performances to nPOLO accuracies obtained in simulations represented in Fig. 2 of the main text. Neural network details are provided for each dataset in the following.

Imagenette: Original dataset consists of 320 by 320 RGB images. Since in nPOLO we use grayscale images we have converted RGB to grayscale. Additionally, the size of images is rescaled to 128 by 128 because of memory considerations for digital networks. The same CNN architecture as LeNet-5[1] is used but fully connected layers differ in

hidden unit size due to the input image size. In this way, hidden unit sizes are 13456, 1024 and 256. For the light CNN, 3 and 6 filters are used in first and second layers of convolutions, respectively. Corresponding hidden unit sizes are: 5046, 256 and 64.

Fashion and Digit MNIST: LeNet 5 is initially designed for the input size of 28 by 28. Since Fashion and Digit MNIST already use the same size, we did not change any hidden units of original LeNet 5. For the light CNN, convolutions filter numbers of 3 and 6 are used in the first and second layers. Consequently, hidden unit sizes are: 96, 64 and 42, respectively.

Supplementary Table 2. *The accuracy comparison of nPOLO framework with digital convolutional neural networks.*

Network Type	Imagenette	Fashion MNIST	Digit MNIST
LeNet 5 (1st Layer: 6 filters, 2nd Layer: 16 filters)	51.06	89.36	98.98
Light CNN (1st Layer: 3 filters, 2nd Layer: 6 filters)	45.7	88.28	98.5
nPOLO	46.3	86.13	96.43

Supplementary Reference

1. LeCun, Y. et al. Backpropagation Applied to Handwritten Zip Code Recognition. Neural Comput. 1, 541–551 (1989).
